# Wide antral circumferential vs. ostial pulmonary vein isolation using pulsed field ablation—the butterfly effect

**DOI:** 10.3389/fcvm.2023.1217745

**Published:** 2023-06-26

**Authors:** Roland R. Tilz, Christian H. Heeger, Julia Vogler, Charlotte Eitel, Marcel Feher, Huong-Lan Phan, Ilias Mushfiq, Sorin S. Popescu, Leonie Zetzsch, Anna Traub, Sascha Hatahet, Kai Mortensen, Karl-Heinz Kuck, Bettina Kirstein

**Affiliations:** ^1^ Department of Rhythmology, University Heart Center Lübeck, University Hospital Schleswig-Holstein, Lübeck, Germany; ^2^ German Center for Cardiovascular Research (DZHK), Partner Site, Lübeck, Germany

**Keywords:** pulmonary vein ablation/isolation, WACA, pulsed field ablation, atrial fibrillation, catheter ablation

## Abstract

**Background:**

Wide antral circumferential ablation (WACA) in comparison to ostial pulmonary vein (PV) isolation (PVI) has been attributed with improved rhythm outcome. We investigated the feasibility, lesion formation, and rhythm outcome of WACA-PVI in comparison to ostial-PVI using pulsed field ablation (PFA).

**Methods:**

Symptomatic atrial fibrillation (AF) patients (69 years, 67% male; 67% paroxysmal AF) were prospectively enrolled into our single-center registry and underwent first-time ostial-PFA or WACA-PFA, *N* = 15 each. In all patients, eight pulse trains (2 kV/2.5 s, bipolar, biphasic, 4× basket/flower configuration each) were delivered to each PV. In WACA-PFA, two extra pulse trains in a flower configuration were added to the anterior and posterior antrum of the PVs. For comparison of PFA lesion size, pre- and post-ablation left atrial (LA) voltage maps were acquired using a multipolar spiral catheter together with a three-dimensional electroanatomic mapping system.

**Results:**

WACA-PFA resulted in a significant larger lesion formation than ostial-PFA (45.5 vs. 35.1 cm^2^, *p* = 0.001) with bilateral overlapping butterfly shape-like lesions and concomitant posterior LA wall isolation in 73% of patients. This was not associated with increased procedure time, sedation dosage, or exposure to radiation. One-year freedom from AF recurrence was numerically higher after WACA-PFA than ostial-PFA (94% vs. 87%) but not statistically significant (*p* = 0.68). No organized atrial tachycardias (ATs) were observed. Ostial-PFA patients more often underwent re-ablation due to recurrent AF episodes.

**Conclusion:**

WACA-PFA is feasible and resulted in significantly wider lesion sets than ostial-PFA. Concomitant posterior LA wall isolation occurred as an epiphenomenon in the majority of patients. The WACA approach was associated with neither increased procedure and fluoroscopy times nor statistically significant differences in 1-year rhythm outcome. ATs were absent.

## Introduction

Atrial fibrillation (AF) is the most common arrhythmia. Pulmonary vein isolation (PVI) is the gold standard for AF ablation procedures and an established, guideline-directed treatment option for symptomatic patients (1).

However, durable PVI by conventional ablation energy sources such as radiofrequency (RF), cryo, or laser energy is highly variable (2) accounting for pulmonary vein (PV) reconnection as the main cause of AF recurrence (3–5).

Wide antral circumferential ablation (WACA) has been investigated as an alternative ablation approach and attributed with an improved rhythm outcome (6). In comparison to ostial-PVI that addresses AF substrates arising from the muscular sleeves of the pulmonary veins (PVs), WACA-PVI also targets arrhythmogenic substrates at the junction between the left atrium (LA) and the posterior LA wall (4, 7–9). As compared to smaller isolation areas, larger isolation areas around the PVs result in a significantly higher PVI success rate (10).

Point-by-point RF energy ablation is the preferred ablation energy source for WACA-PVI. However, RF ablation struggles with potential gap creation, if wider areas and thicker myocardium are targeted. Single-shot devices such as the cryoballoon have been shown to be equally effective to RF energy for treatment of AF (11) but are technically and anatomically limited when WACA is desired.

With pulsed field ablation (PFA), different catheter shapes can be used to create space for more individual lesion set administration. We therefore investigated feasibility, lesion formation, and rhythm outcome during WACA-PVI in comparison to ostial-PVI using PFA.

## Methods

### Patients

Between August and December 2021, consecutive patients with symptomatic AF undergoing pulsed field ablation were prospectively enrolled in our observational, single-center registry. While the first 15 patients received ostial-PFA, all following patients were treated with WACA-PFA. For better comparison, 15 WACA-PFA patients were propensity score matched to the first 15 ostial-PFA patients with respect to age, gender, AF type, and CHA_2_DS_2_VASc score. The registry was approved by the local ethical review board (Lübeck ablation registry ethical review board number: WF-028/15), and all participants provided written informed consent. All investigations were performed in compliance with the ethical standards laid down in the 1964 Declaration of Helsinki and its later amendments.

### Periprocedural management

All patients underwent pre-ablation investigation as per the center's standard of care. Briefly, intracardiac thrombi were ruled out using transesophageal echocardiography. In patients on vitamin K antagonists, the procedure was performed under therapeutic INR values of 2–3. In patients on direct oral anticoagulants (DOACs), the morning dose was omitted on the procedure day. All ablations were performed under analgosedation using propofol, midazolam, and fentanyl following the recommendations of a position paper by the German Cardiac Society (12). Two 8 Fr ultrasound-guided femoral vein punctures were performed. One diagnostic catheter was introduced and positioned deep inside the coronary sinus. A single transseptal puncture (TSP) was performed under fluoroscopic guidance using a modified Brockenbrough technique. After TSP, heparin boluses were administered targeting an activated clotting time of >300 s. An SL1 sheath was used for antegrade LA access. Prior to first pulse delivery, 1 mg atropine was given intravenously to avoid vagal reaction such as sinus arrest or intermittent atrioventricular block.

### Ablation protocol

**Figure 1:**
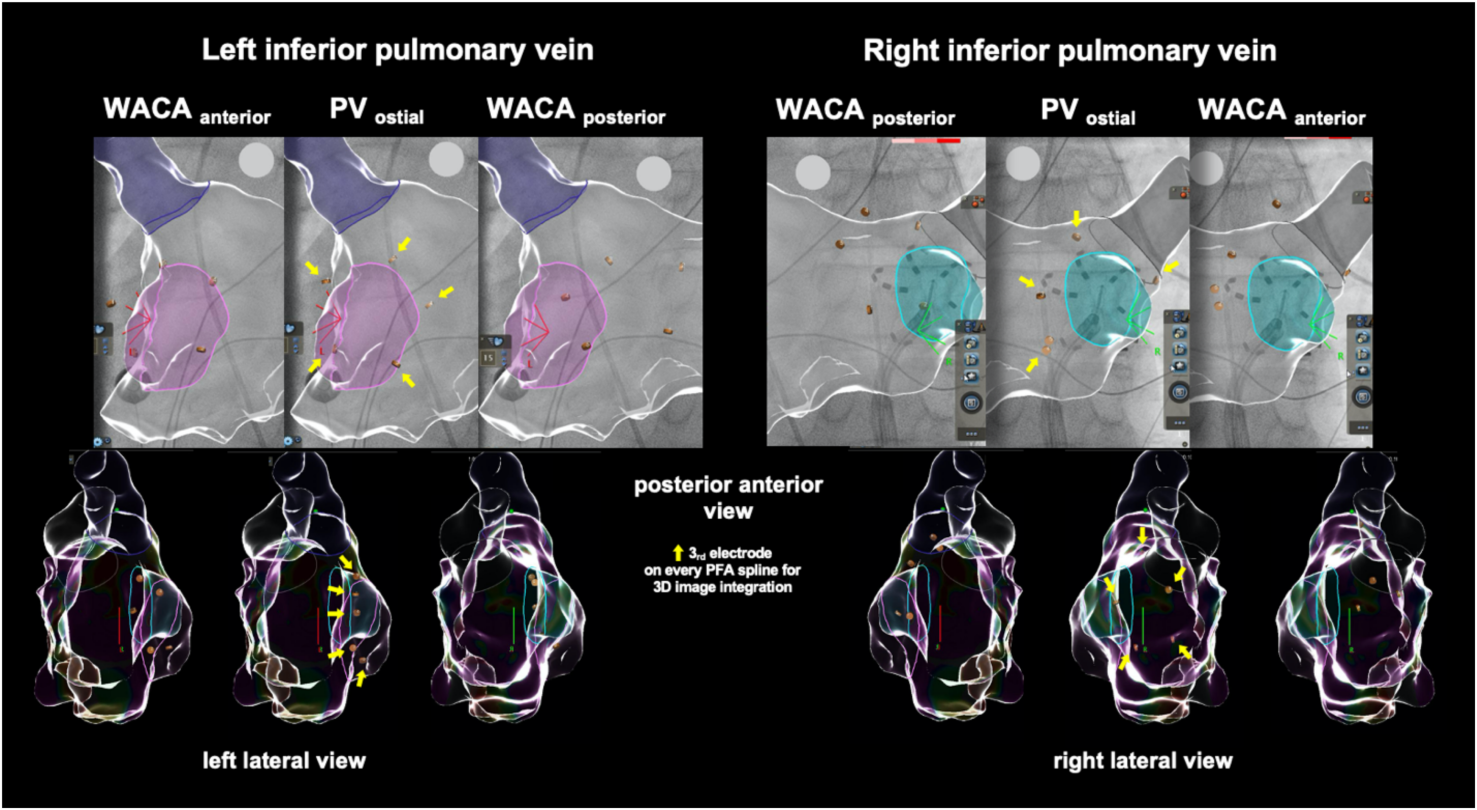
Example of the left atrial model reconstruction in one exemplary patient showing cardiac fluoroscopy and catheter visualization during wide antral circumferential pulsed field ablation (PFA, in flower configuration) of the left and right inferior pulmonary veins when integrated into a 3D navigation system. PFA, pulsed field ablation; PV, pulmonary vein; WACA, wide antral circumferential ablation.

Detailed description of the optimized PFA procedure has been described previously (2). In brief, eight pulse trains (2 kV/2.5 s, bipolar, biphasic, 4× basket and 4× flower configuration each) were delivered to each PV starting on the left-sided veins using the 12-F over-the-wire pentasline PFA catheter (Farawave™; Farapulse-Boston Scientific Inc.). Two extra pulse trains in the flower configuration were added for WACA as per the center's specific protocol. Pulse trains in flower configuration were actively angulated covering a wider anterior and posterior aspect of each PV antrum. Visualization of the PFA catheter angulation for WACA is shown in Figure 1. For implementation, every third electrode on each PFA catheter spline was used for image integration into the 3D navigation system (CARTO 3 V7; Biosense Webster).

### Mapping protocol and lesion measurement

**Figure 2:**
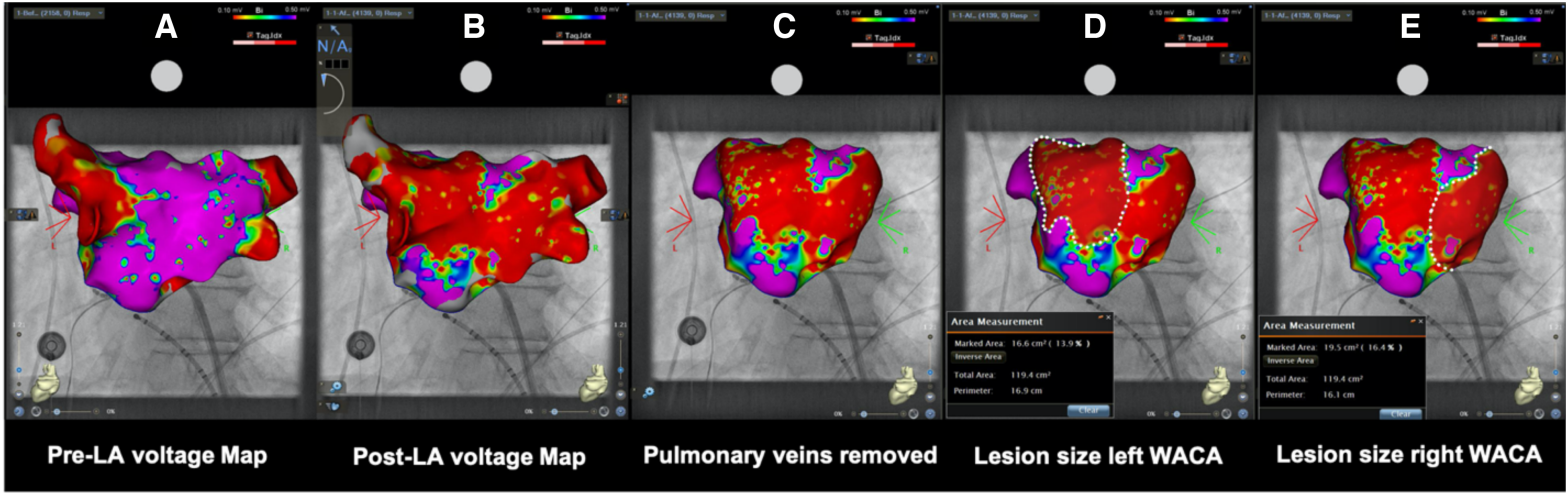
(**A**) Pre- and (**B**) post-ablation left atrial voltage maps in posterior–anterior view of the same patient showing the evaluation of ablation lesion size after wide antral circumferential pulsed field ablation with posterior wall isolation in a “butterfly-like” lesion shape. After anatomical (**C**) removal of the pulmonary veins, (**D**) left- and (**E**) right-sided WACA lesion size was encircled and calculated using dedicated inbuilt software. LA, left atrial/atria; WACA, wide antral circumferential ablation; Bi, bipolar; violet color, vital myocardium; red color, low voltage <0.10 mV indicating ablated myocardium/scar.

Pre- and post-ablation, LA endocardial voltage maps using a three-dimensional electroanatomic reconstruction software (CARTO 3 V7; Biosense Webster or EnsiteX; Abbott) were performed via a multielectrode spiral mapping catheter (Lasso; Biosense Webster, I Advisor™ VL; Abbott Medical). Bipolar peak-to-peak electrogram amplitude <0.5 mV was defined as diseased low voltage signal or scar. No additional touch-up PFA pulses were performed after remapping. On post-ablation maps, the PVs were excluded following their anatomical margins. The ablated area was encircled and measured using vendor-specific software (Figure 2).

### Postprocedural management

A vascular closure device or a figure-of-eight suture and a pressure bandage were used to prevent femoral bleeding. The pressure bandage was removed after 1–4 h depending on the closure technique used. The suture was removed the next day. Following ablation, all patients underwent transthoracic echocardiography immediately, after 2 h, and on the first day post ablation to rule out pericardial effusion. Oral anticoagulants were reinitiated 6 h post ablation and continued for at least 3 months and thereafter according to the CHA_2_DS_2_VASc score. Antiarrhythmic drugs were prescribed for 3 months post ablation, if there was no underlying contraindication, to prevent early AF recurrence during the blanking period. Rhythm follow-up (FU) was based on 24-h Holter ECG directly after the procedure as well as after 3, 6, and 12 months to report the 1-year rhythm outcome.

### Statistical analysis

Continuous variables were tested for normal distribution using the Shapiro–Wilk test and summarized using descriptive statistics. In case of normal distribution, data were presented as mean ± standard deviation (SD), or as median and interquartile range (IQR) with first quartile (Q1), third quartile (Q3). Comparison of continuous data was performed using Student's *t*-test if normally distributed, or with the Wilcoxon signed-rank test.

Categorical data are reported as absolute (*n*) and relative frequencies (%) and were compared using the chi-square test or Fisher's exact test (small expected frequencies).

For group comparisons, normality of data was verified with the use of box plots and Kolmogorov–Smirnov normality test. For normally distributed data, comparisons were performed using Student's *t*-test for independent groups. In cases where data were not normally distributed, the nonparametric Wilcoxon rank sum test was used.

Results were expressed in terms of *p*-values. Exact two-sided statistical significance was considered for a *p*-value <0.01. All calculations were performed with the statistical analysis software DATAtab (DATAtab e.U., Graz, Austria).

## Results

### Patient characteristics

Thirty patients with predominantly clinical symptomatic paroxysmal AF, a normal left ventricular ejection fraction, and a low burden of comorbidities (median CHA_2_DS_2_VASc score of 2) were prospectively enrolled into the registry. Prior pharmacological or electrical rhythm control using antiarrhythmic drugs or electrical cardioversion had been attempted in 43% and 37% of patients, respectively. Oral anticoagulation with a DOAC was established in the majority of patients. Baseline characteristics did not differ between both groups. Detailed patient baseline characteristics are shown in Table 1.

**Table 1:** Baseline patient characteristics.

	All (*N* = 30)	Ostial-PFA (*N* = 15)	WACA-PFA (*N* = 15)	*p*-value
Demographics				
Age (years)	69 (55, 72)	69 (54, 71)	68 (59, 73)	0.388
Gender (male, %)	67	67	67	1
BMI	26 (23, 29)	25 (24, 28)	27 (23, 30)	0.767
AF type (paroxysmal, %)	67	67	67	1
AF diagnosis (months)	11 (3, 26)	9 (3, 31)	11 (4, 25)	0.492
EHRA class IIa and IIb (%)	76	73	80	0.267
CHA_2_DS_2_VASc score	2 (1, 3)	2 (1, 3)	3 (2, 4)	0.322
Cardioversion (%)	37	27	47	0.256
AAD treatment (%)	43	47	40	0.713
Anticoagulation				0.067
None (%)	13	27	0	
DOAC (%)	77	60	93	
Vitamin K antagonist (%)	10	13	7	
Comorbidities				
aHT (%)	60	53	67	0.456
DM (%)	7	0	13	0.143
CM (%)	20	13	27	0.361
CAD (%)	7	0	13	0.143
Stroke/TIA/embolism (%)	13	13	13	1
OSAS (%)	3	0	7	0.309
CKD (%)	7	0	13	0.143
Echocardiography				
LVEF (%)	55 (50, 60)	58 (55, 60)	52 (41, 55)	0.035
LA (dilated, %)	33	33	42	0.656
MR (I–II, %)	40	40	46	0.743
LAA flow (m/s)	0.6 ± 0.3	0.7 ± 0.2	0.5 ± 0.3	0.053

WACA, wide antral circumferential ablation; BMI, body mass index; AF, atrial fibrillation; AAD, antiarrhythmic drug; DOAC, direct oral anticoagulant; aHT, arterial hypertension; DM, diabetes mellitus; CM, cardiomyopathy; CAD, coronary artery disease; TIA, transient ischemic attack; OSAS, obstructive sleep apnea syndrome; CKD, chronic kidney disease; LVEF, left ventricular ejection fraction, LA, left atria; MR, mitral regurgitation; LAA, left atrial appendage.

Continuous data are given in median and interquartile range (Q1, Q3) and categorical data are given in percentage of *N*.

### Procedural characteristics

All PFA procedures were performed by two highly experienced EP operators. A median of 8 (IQR 8, 8) and 10 (IQR 9, 12) pulse trains per PV for ostial and WACA-PFA were applied. All PVs were isolated at the end of the procedure. The WACA-PFA approach was not associated with a significant increase in procedure time, sedation dosage, or exposure to radiation. Procedural data are depicted in Table 2.

**Table 2:** Procedural characteristics and rhythm outcome.

	All (*N* = 30)	Ostial-PFA (*N* = 15)	WACA-PFA (*N* = 15)	*p*-value
PFA procedure				
Procedure time (min)	66 (60, 74)	65 (50, 73)	66 (60, 73)	0.29
Fluoroscopy time (min)	11 (10, 15)	11 (9, 13)	13 (10, 16)	0.26
Dose area product (cGv/cm^2^)	396 (304, 623)	413 (269, 603)	382 (339, 637)	0.71
Contrast medium (ml)	40 (40, 50)	40 (30, 50)	40 (40, 50)	0.35
Heparin (IEx1000)	17 (13, 20)	17 (14, 20)	15 (12, 20)	0.35
Propofol (1% mg)	575 (500, 748)	500 (470, 745)	600 (500, 755)	0.78
Midazolam (mg)	2.5 (2, 4)	2.5 (2, 4)	2.5 (2, 3)	0.25
Fentanyl (mg)	50 (50, 50)	50 (50, 63)	50 (50, 50)	0.71
Left atrial mapping				
Catheter dwell time (min)	19 (15, 24)	19 (16, 26)	18 (16, 21)	0.47
PFA catheter size 31 mm (%)	33	20	47	0.09
Pre-mapping point density	2,344 ± 1,202	2,178 ± 1,515	2,498 ± 839	0.48
Post-mapping point density	2,819 ± 1,534	2,719 ± 1,521	2,905 ± 1,592	0.76
PFA lesion size (cm^2^)	40.4 ± 9.3	35.1 ± 7.2	45.5 ± 8.3	0.001^a^
LA wall isolation (*n*, %)		0 (0)	11/15 (73)	0.001^a^
Rhythm outcome				
Freedom from arrhythmia (%)	90	87	94	0.68
Re-ablation (*n*, %)	2/30 (7%)	2/15 (13%)	0 (0%)	0.14

PFA, pulsed field ablation; WACA, wide antral circumferential ablation; LA, left atrial.

Continuous data are given in median and interquartile range (Q1, Q3) and categorical data are given in percentage of *N*.

^a^ Statistically significant.

### Mapping characteristics and lesion metrics

**Figure 3:**
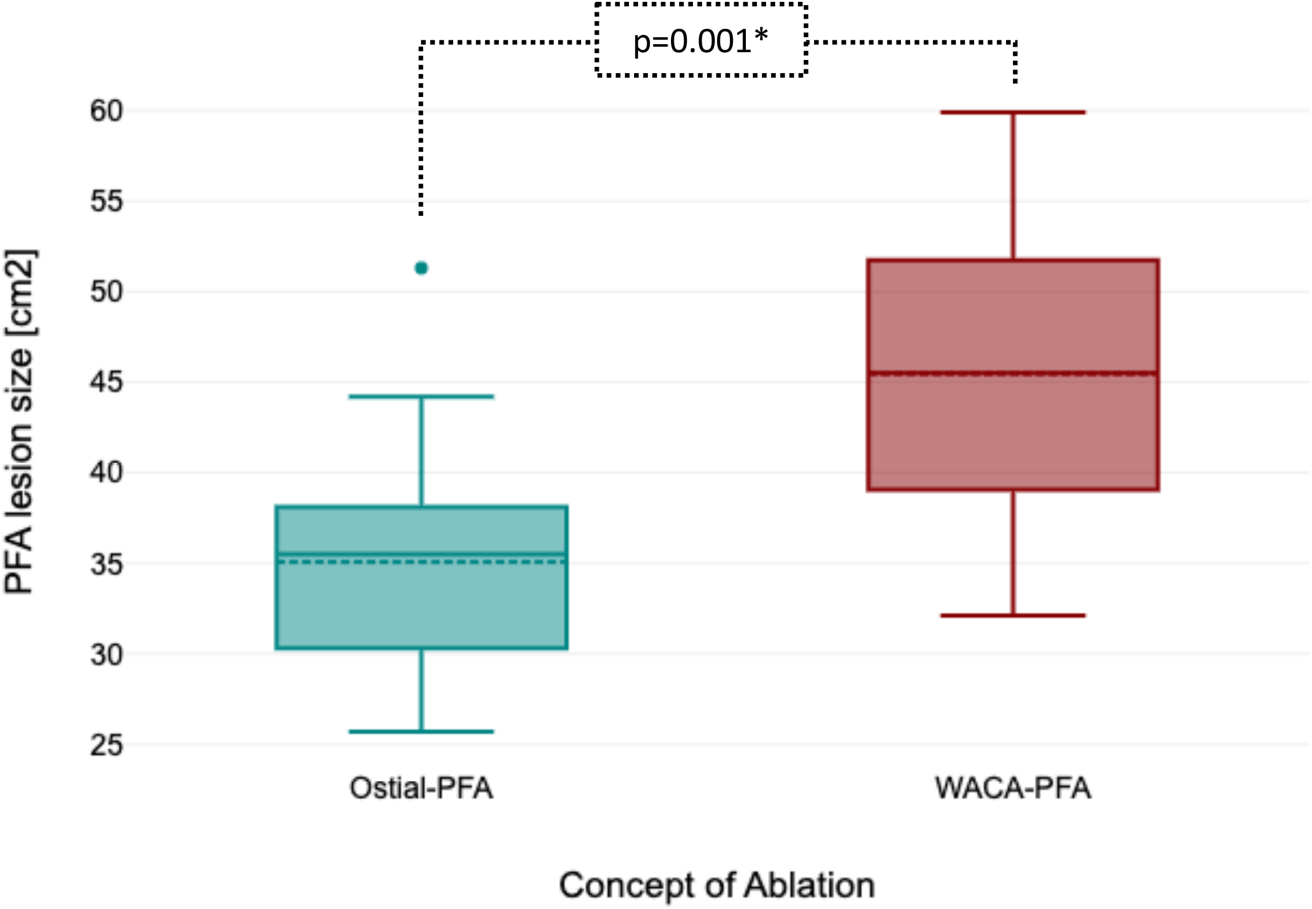
Boxplot showing significant larger ablation lesion size between ostial-PFA and WACA-PFA. PFA, pulsed field ablation; WACA, wide antral circumferential ablation.

**Figure 4:**
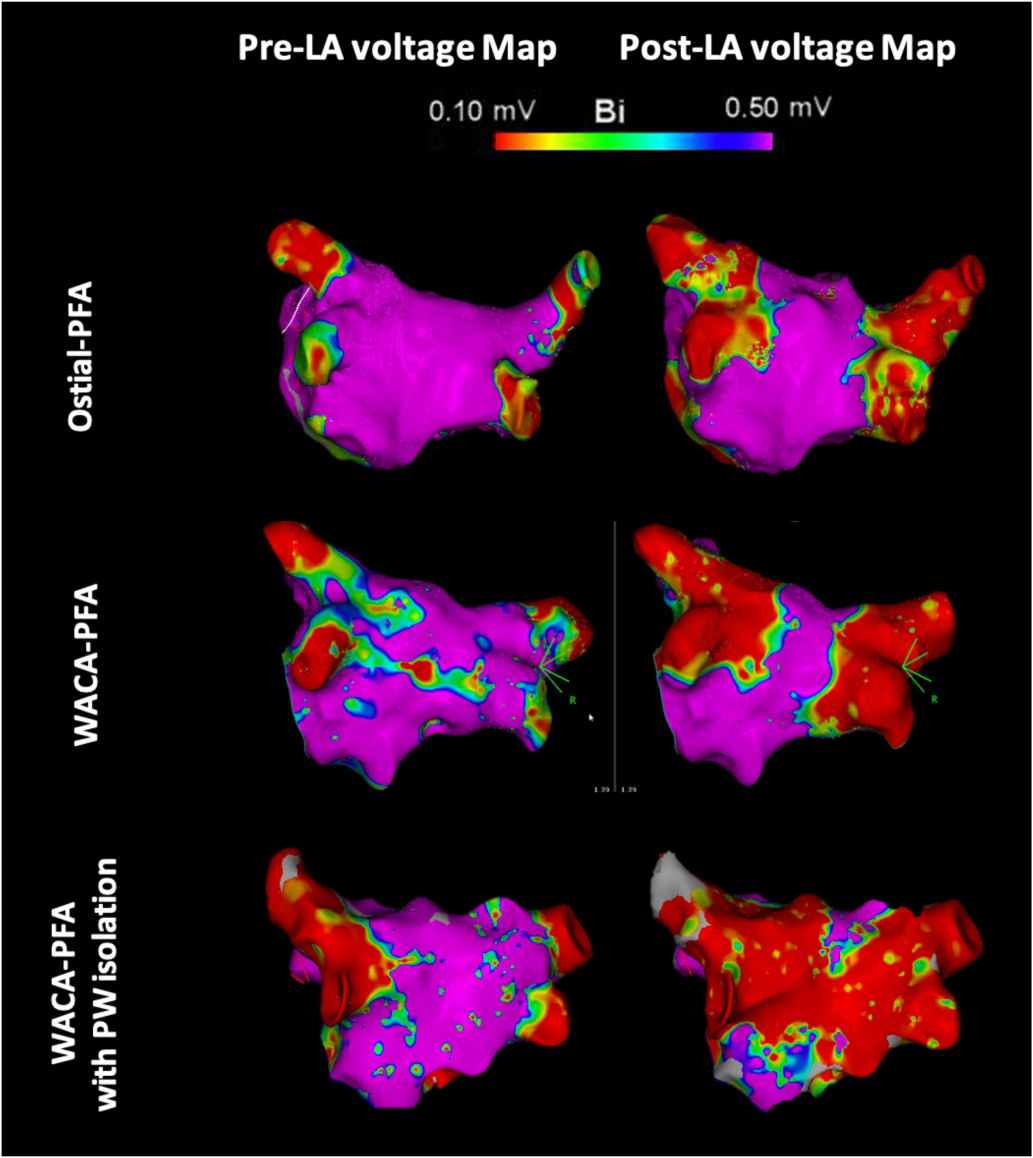
Pre- and post-ablation left atrial voltage maps in posterior–anterior view showing different lesion formation in ostial and wide antral circumferential pulsed field ablation with and without posterior wall isolation in a “butterfly-like” lesion shape. LA, left atrial/atria; PFA, pulsed field ablation; WACA, wide antral circumferential ablation; PW, posterior wall; Bi, bipolar; violet color, vital myocardium; red color, low voltage <0.10 mV indicating ablated myocardium/scar.

Left atrial pre- and post-mapping point density did not differ between both groups. In the WACA-PFA group, a trend to usage of a smaller ablation catheter size was noted. WACA-PFA resulted in significantly larger lesion formation (45.5 ± 8.3 cm^2^) than ostial-PFA (35.1 ± 7.2 cm^2^), *p* = 0.001 as shown in Figure 3 with overlapping bilateral lesion sets and consecutive posterior LA wall isolation in 11/15 (73%) patients. In Figure 4, we provide examples highlighting the different lesion sets on pre- and post-ablation LA voltage maps for ostial-PFA, WACA-PFA, and WACA-PFA with an overlapping bilateral lesion set and electrical posterior LA wall isolation—the so-called “butterfly effect.”

### Adverse events

In the ostial-PFA group, four patients presented with transient phrenic nerve stunning—with full recovery the next day. One patient experienced acute postprocedural hemoptysis with no obvious bleeding source in the bronchoscopy, with spontaneous cessation without need for transfusion.

In the WACA-PFA group, one of each of the following events occurred either acute or during the further clinical follow-up: hemodynamically stable pericardial effusion recurrent under conservative therapy, groin hematoma and deep vein thrombosis at the venous access sight, decompensated heart failure with preserved ejection fraction despite sinus rhythm.

### Rhythm outcome

Completeness of Holter ECG recordings were 40% for all, 73% for at least three, 97% for at least two, and 100% for one Holter. Freedom from arrhythmia recurrence was 87% (*N* = 2/15) after ostial-PFA and 94% (*N* = 1/15) after WACA-PFA, which was not statistically different (*p* = 0.68). Both patients in the ostial-PFA group had recurrent symptomatic atrial arrhythmia episodes and underwent re-ablation. The patient in the WACA-PFA group experienced only one paroxysmal episode several days after the end of the blanking period and therefore was not scheduled for re-ablation so far. We did not observe organized atrial tachycardia (AT).

## Discussions

### Main findings

In our prospective registry, WACA-PFA in comparison to ostial-PFA resulted in significantly wider lesion sets. Using the currently available PFA catheter, we observed bilateral overlapping lesion sets with concomitant posterior LA wall isolation as an epiphenomenon in the majority of patients. This was not associated with prolonged procedure or fluoroscopy times. Rhythm outcome after 1 year was numerically better in the WACA-PFA group but was not statistically significant. However, only ostial-PFA patients underwent re-ablation due to recurrent episodes.

PFA is a novel ablation technology with promising results over existing ablation energies due to its unique myocardial tissue specificity (2, 13). Early preclinical and clinical data reported improved acute and chronic PVI rates up to 96% (14) without regression of the isolated area size between the acute and chronic phase (>75 days post-PFA) (15). On pathohistological assessment and gross anatomy models, PFA resulted in more homogeneous and transmural ablation lesion formation with less coagulation in comparison to RF energy, serving as a prerequisite for durable PVI (2, 16–18). Evaluation with high-density voltage mapping confirmed these findings. Sharp delineated lesion boundaries between scar and healthy tissue were described (19, 20). When lesion pattern between RF, cryo, and PFA energy on the posterior aspect of the PV antrum were compared against each other by electroanatomical mapping, PFA lesions resulted in an RF-like pattern, also encompassing the carina level of the ipsilateral PVs, which was left with notched-like areas of normal voltage when the cryoballon was used (21). In comparison to previously reported data on lesion size, ostial-PFA alone resulted in a larger ablated area than cryoballon ablation (35.1 vs. 28.9 cm^2^) (22).

Recently published data on high-density voltage mapping revealed the anterior and superior parts of both sided PVs as susceptible weak spots for gap formation after PFA (20). The authors attributed this to the over-the wire catheter design, which is prone to alignment to the posterior wall after transseptal puncture. Respecting this limitation, our modified WACA-PFA approach with active anterior and posterior catheter angulation resulted in larger lesion set formation. Subsequently, important arrhythmogenic foci on the anterior ridge, the carina level, and at the posterior aspects of the PV antrum were addressed. However, careful handling, tissue proximity, and experience in interventional left atrial procedures are needed to avoid collateral damage to the anterior and posterior LA wall, as seen in our population with the latter. This may lead to potentially proarrhythmogenic isthmus creation on the posterior wall and has been associated with the development of roof-dependent ATs. In our cohort, we did not observe organized ATs. In most of our WACA-PFA patients, bilateral overlapping areas of low voltage were assessed on acute electroanatomical remapping. This might have prevented the development of ATs as the type of arrhythmia recurrence. Overall, a more reliable catheter integration into available three-dimensional mapping systems would be desirable to better understand the site of energy delivery.

The variable opening degree and shapes of the current PFA catheter (from basket to olive to donut- and flower-like formation) enable individual lesion application, on the one hand, but can result in different pulsed field characteristics with divergent lesion characteristics, on the other hand. We therefore used the currently recommended and evaluated flower shape of the PFA catheter for WACA lesion application. Overall, this approach resulted in a high percentage of freedom from AF recurrence after 1 year with both approaches. This is in line with recently published 1-year outcome data reporting 78.5% freedom from atrial arrhythmia after PFA (23). However, patients with ostial-PFA had multiple AF recurrences and therefore underwent re-ablation. Further evaluation of the WACA-PFA approach in a larger number of patients is needed.

### Limitations

Our data present a single-center registry experience and encompass a small number of patients with early AF stages and low rate of comorbidities. No randomization or comparison to other ablation energy sources was performed. Data are only applicable to the vendor-specific PFA catheter design and technology. Potential bias might result from using variable PFA catheter sizes. No data on serum concentrations of highly sensitive troponin I as a surrogate for larger lesion extent were evaluated. Routine PFA catheter integration into the three-dimensional mapping system was not performed due to instable performance issues.

## Conclusions

In our prospective registry, WACA-PFA in comparison to ostial-PFA was feasible. WACA-PVI resulted in significantly wider lesion sets and a bilaterally overlapping lesion formation on the posterior LA wall, the so-called “butterfly effect.” Concomitant posterior LA wall isolation occurred as an epiphenomenon in the majority of patients. This approach was not associated with a significant increase in the procedure time, sedation dosage, or exposure to radiation nor differences in rhythm outcome, especially ATs were absent. However, only ostial-PFA patients underwent re-ablation procedures due to recurrent symptomatic AF episodes. This needs to be evaluated further on longer follow-up and in a larger cohort of patients.

## Data Availability

The datasets presented in this article are not readily available because non-digital data supporting this study are curated at the Study Center of the Department of Rhythmology, University Hospital Schleswig-Holstein, Germany. Requests to access the datasets should be directed to UKSH Campus Lübeck, Studienzentrale Rhythmologie, https://www.uksh.de/rhythmologie-luebeck/Forschung+_+Lehre/Studienzentrale.html.
