# Supplementary figures and images for: Wide antral circumferential vs. ostial pulmonary vein isolation using pulsed field ablation—the butterfly effect
Source: Front Cardiovasc Med. 2023 Jun 26;10:1217745. doi: 10.3389/fcvm.2023.1217745 (PMC10331428; doi:10.3389/fcvm.2023.1217745)

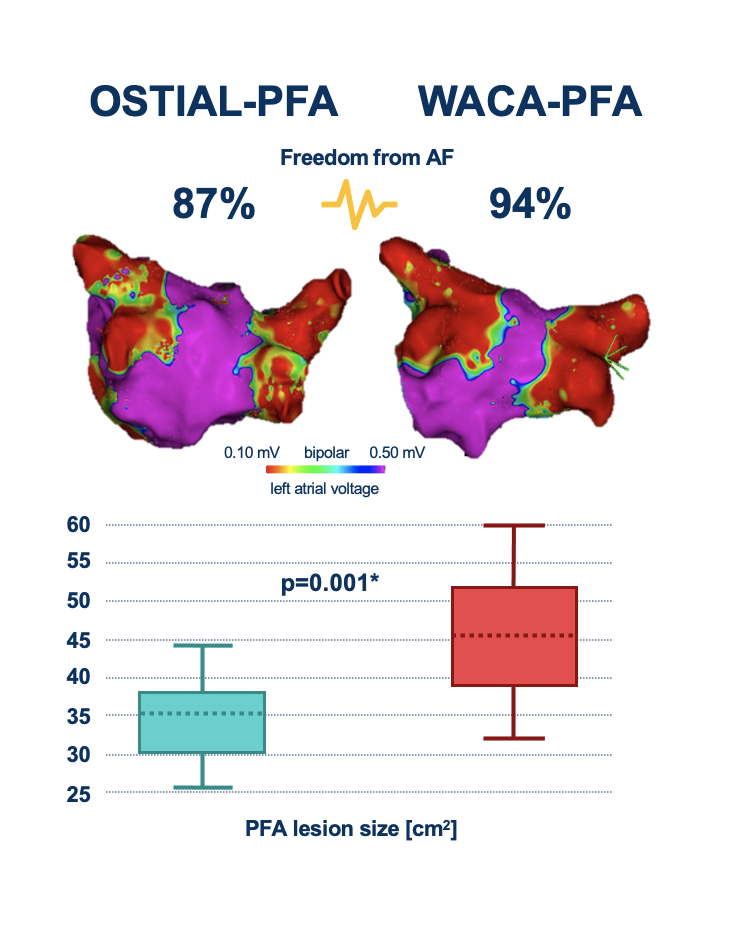

Supplement: Supplementary file 1 [file Image1.png]
